# Antiretroviral treatment and quality of life in Africans living with HIV: 12-month follow-up in Burkina Faso

**DOI:** 10.7448/IAS.16.1.18867

**Published:** 2013-12-20

**Authors:** Antoine Jaquet, Franck Garanet, Eric Balestre, Didier K. Ekouevi, Jean Claude Azani, René Bognounou, Elias Dah, Jean Charlemagne Kondombo, François Dabis, Joseph Drabo, for the IeDEA West Africa collaboration

**Affiliations:** 1 Université Bordeaux, ISPED, Centre INSERM U897 – Epidémiologie-Biostatistique, F-33000 Bordeaux, France; 2 INSERM, ISPED, Centre INSERM U897 – Epidémiologie-Biostatistique, F-33000 Bordeaux, France; 3 Service de Médecine interne, Centre Hospitalier Universitaire Yalgado Ouedraogo (CHU-YO), Ouagadougou, Burkina Faso; 4 PACCI, Centre Hospitalier Universitaire (CHU) de Treichville, Abidjan, Cote d'Ivoire; 5 Centre médical associatif African Association Solidarity (AAS), Ouagadougou, Burkina Faso; 6 Centre médical avec antenne chirurgicale de Pisssy, Ouagadougou, Burkina Faso

**Keywords:** quality of life, HIV/AIDS, antiretroviral treatment, Burkina Faso, sub-Saharan Africa

## Abstract

**Introduction:**

The scale-up of highly active antiretroviral therapy (HAART) has led to a significant improvement in survival of the HIV-positive patient but its effects on health-related quality of life (HRQOL) are less known and context-dependent. Our aim was to assess the temporal changes and factors associated with HRQOL among HIV-positive adults initiating HAART in Burkina Faso.

**Methods:**

HIV-positive people initiating HAART were prospectively included and followed over a one-year period in three HIV clinics of Ouagadougou. HRQOL was assessed at baseline and at each follow-up visit using physical (PHS) and mental (MHS) summary scores derived from the Medical Outcome Study 36-Item short-form health survey (MOS SF-36) questionnaire. Toxicity related to HAART modification and self-reported symptoms were recorded during follow-up visits. Determinants associated with baseline and changes in both scores over a one-year period were assessed using a mixed linear model.

**Results:**

A total of 344 patients were included. Their median age at baseline was 37 years [interquartile range (IQR) 30–44] and their median CD4 count was 181 cells/mm^3^ (IQR 97–269). The mean [standard deviation (SD)] PHS score increased from 45.4 (11.1) at baseline to 60.0 (3.1) at 12 months (*p*<10^−4^) and the mean (SD) MHS score from 42.2 (8.7) to 43.9 (3.4) (*p*<10^−2^). After one year of treatment, patients that experienced on average two symptoms during follow-up presented with significantly lower PHS (63.9) and MHS (43.8) scores compared to patients that presented no symptoms with PHS and MHS of 68.2 (*p*<10^−4^) and 45.3 (*p*<10^−3^), respectively.

**Discussion:**

The use of HAART was associated with a significant increase in both physical and mental aspects of the HRQOL over a 12-month period in this urban African population. Perceived symptoms experienced during follow-up visits were associated with a significant impairment in HRQOL. The appropriate and timely management of reported symptoms during the follow-up of HAART-treated patients is a key component to restore HRQOL.

## Introduction

The rapid and high uptake of highly active antiretroviral therapy (HAART) over the past decade has led to a significant improvement in survival of the HIV-positive patient in West Africa [1–3]. As life expectancy of HIV-positive people continues to increase, their health-related quality of life (HRQOL) is now also becoming an important issue for the sustainability of HAART programs. Indeed, to be sustainable, HAART needs to be accepted by HIV-positive patients over a long period of time. Thus, HRQOL could be a key indicator for tracking the long-term acceptability and efficiency of HAART in the context of a chronic condition such as HIV infection. In this perspective, HAART programs in West Africa need now to incorporate monitoring systems that measure both the effects of HAART on clinical markers and on overall wellbeing, including HRQOL.

HRQOL is a multidimensional construct that includes global health perspectives, functional status, biological and physical variables, individual and environmental characteristics and general perceptions [4]. Many instruments have been developed to quantify HRQOL. The Medical Outcome Study 36-Item short-form health survey (MOS SF-36) questionnaire is characterized by the fact that it explores two dimensions. The first dimension measures the disease consequences on physical quality of life by assessing physical and functional limitations. The second dimension addresses changes in the emotional state of patients, with an emphasis on mood, depression and life satisfaction [5, 6].

Since the advent of HAART, a significant improvement of HRQOL has been reported over time in treatment-naïve patients initiating HAART [7, 8]. However, these reports mainly conducted in controlled clinical trials focused on HIV-positive patients from high-income settings. Early reports from Southern and Eastern African countries have prospectively explored the impact of HAART on HRQOL of HIV-positive people over a 12-month period, showing an overall positive effect [9, 10]. Authors have also highlighted a potential negative effect of adverse drug reactions on HRQOL evolution after HAART initiation but their assessment of adverse events relied only on toxicity associated with treatment modification. None of them have taken into account patient's perceived symptoms or effective adherence to HAART [11, 12]. Moreover, these findings reported at the beginning of the HAART era are hardly extendable to West African countries with a very different socioeconomic and cultural context. Burkina Faso is a country with a generalized HIV epidemic, which has one of the highest HAART coverage in West Africa. Indeed, by the end of 2010, more than 31,000 (49%) of the 64,000 estimated people living with HIV were receiving HAART in this country [13]. This report aims at assessing the temporal changes and factors associated with HRQOL evolution over a 12-month period in HIV-positive adults initiating HAART in the urban area of Ouagadougou, Burkina Faso.

## Methods

### Study design and population

From April to December 2010, three HIV clinics located in the urban area of Ouagadougou, one in the university hospital, one in a general public hospital and one run by a non-governmental organization, systematically and consecutively proposed their participation to all HIV-positive patients initiating HAART. Patients were eligible for the present study if they were HAART-naïve, aged ≥18 years and if they gave their informed consent to participate in the present study.

### Baseline visit

In the three participating clinical sites, healthcare providers were in charge to administer a baseline standardized questionnaire to patients initiating HAART during face-to-face interviews. This questionnaire assessed patient's characteristics including gender, economic resources defined by their monthly income and level of formal education (no school versus primary school or secondary school and over). Alcohol use was reported using the Alcohol Use Disorder Identification Test (AUDIT) that measured alcohol use and its impact on health during the past 12 months [14]. Tobacco use was defined according to three categories: current smoker, past smoker and ever smoker as described in a previous report from West Africa [15]. Disclosure was defined as having revealed their HIV status or not to relatives or friends. Discrimination was defined as a binary variable in response to the following formulation: did you felt neglect, contempt or did you experience other behaviours that have taken away relatives, friends or healthcare providers because of your HIV status. After completing these interviews, healthcare providers were in charge to report from their medical charts information related to patient's baseline clinical characteristics including CD4 cell count (cells/mm^3^) and clinical stage according to World Health Organization (WHO) classification at HAART initiation.

#### Health-related quality of life

To assess the HRQOL, the MOS SF-36 questionnaire was administered. The choice of this tool relied on several arguments. First, a validated French version, the official language in Burkina Faso, is available [16]. Second, it is a widely used generic questionnaire, which has been already administered in the specific context of HIV infection in France and also in sub-Saharan Africa [17–19]. The MOS SF-36 is composed of 36 items assessing eight health concepts: limitations in physical activities because of health problems, limitations in social activities because of physical or emotional problems, limitations in usual role activities because of physical health problems, bodily pain, general mental health (psychological distress and wellbeing), limitations in usual role activities because of emotional problems, vitality (energy and fatigue) and general health perceptions.

Scores for each concept of the HRQOL were derived from responses to the 36 items of the MOS SF-36 and transformed into a scale ranging from 0 to 100, with higher score values corresponding to better HRQOL. Two summary scores were then derived from these scales, the physical health summary (PHS) score and the mental health summary (MHS) score. These summary scales were obtained by using a method that standardizes the scores so that the mean is 50 and the standard deviation is 10 [20]. We adapted this version of the questionnaire by piloting the administration of the original form and adding some minor explanations in specific items. After this pilot phase, all health workers involved in the study were trained together on the administration of the locally adapted MOS SF-36 during a one-day session.

### Follow-up visits

As part of their usual follow-up, HIV-positive patients initiating HAART were scheduled for follow-up visits at month three, six and twelve. During these follow-up visits, healthcare providers were in charge to administer the dedicated questionnaire assessing HRQOL using the same MOS SF-36 survey form.

#### Drug-related toxicity and number of self-reported symptoms

Any HAART modification, as well as its causes including drug-related toxicity that occurred during the follow-up period, was documented at each follow-up visits. In addition, patients were also asked about the occurrence of 18 listed disease-related symptoms during the previous four weeks. This list was developed by Justice *et al*. and included symptoms such as dizziness, memory loss, nausea/vomiting, muscle/joint pain, diarrhoea, abdominal pain, headaches, sleep trouble as described elsewhere [21]. Symptoms of this list have been previously used as a proxy to capture undiagnosed drug-related toxicity in clinical trials conducted in industrialized countries [22, 23]. Indeed, relying only upon physician's assessment to capture treatment-related adverse events may underestimate toxicity perceived by patients [23, 24]. The presence of any of these symptoms during follow-up visits was summed for each patient in order to provide a quantitative assessment of the total number of perceived symptoms.

#### Adherence to HAART

The questionnaire also included a four-day recall on adherence to HAART based on the AIDS Clinical Trials Group (ACTG) follow-up questionnaire for adherence to antiretroviral medications [25]. Adherence was coded as a dichotomous variable, where patients <95% adherent during the previous four days were considered non-adherent (based on previous studies suggesting that ≥5% missed doses was associated with poorer virological outcomes) [26]. For the present analysis, patients were defined as non-adherent to HAART in the model if they were identified non-adherent according to the previous definition during at least one of the three follow-up visits.

### Statistical analysis

We used a Chi^2^ test to compare baseline qualitative characteristics and non-parametric test (Kruskal-Wallis) to compare baseline quantitative characteristics according to gender. Matched paired *t*-tests were used to compare the observed PHS and MHS change as well as their respective eight domains from baseline to month 12.

#### HRQOL modelling

To examine factors associated with HRQOL, we chose to assess changes over a 12-month period of the two summary scores (PHS and MHS). We used two separate linear mixed models (LMM) to determine factors associated with a PHS or MHS change over a 12-month period. Both PHS and MHS scores were normally distributed.

We used LMM with one slope and an intercept. The variance of random effects on the intercept and on the slope was tested and was not significantly different to 0 (unstructured and diagonal covariance matrix was both tested for random effects). Thus, only fixed effects were included in the two LMMs. The following adjustment variables were investigated for both models: age, education, disclosure, discrimination, perceived symptoms, drug-related treatment modification, baseline WHO clinical stage, baseline CD4 cell count, HIV clinic, antiretroviral regimen as well as adherence to antiretroviral treatment. Variables that were statistically associated with HRQOL with a *p*-value ≤0.25 in the univariable analysis were selected in the multivariable analysis. The two final LMM were performed by using a manual descending method and potential confounders were investigated. A *p*-value≤0.05 was considered as statistically significant in the final models. We reported the mean PHS and MHS scores at baseline as well as the mean PHS and MHS changes for a one-month period with their 95% confidence intervals (CIs). Finally, we used the parameters of the multivariable LMM to estimate mean PHS and MHS scores at 12 months according to the variables selected in the models. Residual homoscedasticity and residual normality were graphically verified. All statistical analyses were performed with SAS version 9.2 (SAS Institute Inc., Cary, NC, USA).

## Results

A total of 364 HIV-positive patients initiated HAART during the study period in the three participating clinics. Of these, 344 (94.5%) were included and assessed at baseline, 13 (3.6%) patients refused to participate in the present study and seven (1.9%) patients were excluded as they had taken mono or dual-therapy prior to HAART initiation. Of the 344 patients included with a baseline HRQOL assessment, 299 (86.9%) were reassessed for HRQOL during their follow-up visits at three months, 274 (79.6%) at six months and 265 (77.0%) at 12 months. Six (1.7%) patients were transferred to another clinic during the first year of follow-up and 13 (3.8%) patients were reported as dead.

### Baseline characteristics

Patients with a baseline HRQOL assessment had a median age of 37 years [interquartile range (IQR) 30–44] and 73.8% were women. Their median CD4 count at HAART initiation was 181 cells/mm^3^ (IQR 97–269) and 138 (40.0%) were diagnosed with a stage III or IV classifying disease according to WHO classification. Of the 344 participants that initiated HAART, 316 (91.9%) were below the treatment initiation threshold defined by a CD4 count ≤350 cells/mm^3^ and/or the presence of a severe HIV infection defined by a stage 3 or 4 clinical condition according to the WHO recommendations. Additional socio-demographic and clinical characteristics of this sample of HAART initiators are summarized in Table 1.

**Table 1:** Baseline characteristics of patients initiating HAART according to gender in Ouagadougou, Burkina Faso. The IeDEA West Africa collaboration, 2010–2011

	Women (N=254)	Men (N=90)	*p*	Total (N=344)
Age, median (IQR)	35 (30–42)	41 (34–49)	<10^−4^	37 (30–44)
Monthly income (USD), *n* (%)			<10^−4^	
<80	173 (68.1)	42 (46.7)		215 (62.5)
≥80–160	38 (15.0)	32 (35.6)		70 (20.3)
≥160	43 (16.9)	16 (17.8)		59 (17.2)
Formal education, *n* (%)			0.23	
No school	108 (42.5)	30 (33.3)		38 (40.1)
Primary school	69 (27.2)	25 (27.8)		94 (27.3)
Secondary and over	77 (30.3)	35 (38.9)		112 (32.6)
Smoking status, *n* (%)			<10^−4^	
No smoker	242 (95.3)	59 (65.6)		301 (87.5)
Present/past smoker	12 (4.7)	31 (34.4)		43 (12.5)
Alcohol use,^a^ *n* (%)			0.01	
No	192 (75.6)	56 (62.2)		248 (72.1)
Yes	62 (24.4)	34 (37.8)		96 (27.9)
Disclosure,^b^ *n* (%)			0.65	
No	77 (30.3)	25 (27.8)		102 (29.7)
Yes	177 (69.7)	65 (72.2)		242 (70.3)
Discrimination,^c^ *n* (%)			0.63	
No	230 (90.6)	83 (92.2)		313 (91.0)
Yes	24 (9.4)	7 (7.8)		31 (9.0)
HIV clinic, *n* (%)			0.55	
University hospital	102 (40.2)	38 (42.2)		140 (40.7)
General hospital	56 (22.0)	15 (16.7)		71 (20.6)
NGO	96 (37.8)	37 (41.1)		133 (38.7)
Body mass index (kg/m2), median (IQR)	20.7 (18.4–23.0)	19.6 (17.3–22.1)	0.02	20.5 (18.0–22.7)
CD4 count (cells/mm^3^), median (IQR)	193 (98–280)	162 (96–213)	0.02	181 (97–270)
Clinical stage,^d^ *n* (%)			0.05	
I or II	160 (63.0)	45 (50.0)		205 (59.6)
III or IV	91 (35.8)	42 (46.7)		133 (38.7)
Missing values	3 (1.2)	3 (3.3)		6 (1.7)
First-line regimen, *n* (%)			0.12	
AZT+3TC+NVP	86 (33.9)	29 (32.2)		115 (33.4)
D4T+3TC+NVP	76 (29.9)	15 (16.7)		91 (26.5)
AZT+3TC+EFV	47 (18.5)	26 (28.9)		73 (21.2)
TDF-based regimens	23 (9.1)	11 (12.2)		34 (9.9)
D4T+3TC+EFV	13 (5.1)	6 (6.7)		19 (5.5)
Other regimens	9 (3.5)	3 (3.3)		12 (3.5)

a Any alcohol use declared during the past 12 months

b to relatives or friends

c from relatives, friends or healthcare providers

d clinical stage according to World Health Organization classification. IQR=interquartile range; NGO=non-governmental organization; AZT=zidovudine; D4T=stavudine 3TC=lamivudine; NVP=nevirapine; EFV=efavirenz; TDF=tenofovir.

### Follow-up characteristics

During the first 12 months of follow-up, 73 treatment modifications were reported in 71 patients. A total of 30 drug-related toxicities were reported and represented the main (41.1%) reason of treatment modification: 12 (41.4%) cutaneous reactions, nine (31.0%) peripheral neuropathy, three (10.3%) gastrointestinal disorders, two (9.9%) lipodystrophy, two (6.9%) elevated transaminases, one (3.4%) central nervous disorders and one (3.4%) renal toxicity.

A median number of 2 (IQR 0–6) self-reported symptoms were declared by patients during their follow-up. The distribution of reported symptoms among patients that attended at least one follow-up visit was as follows: 149 (11.9%) changes in the body image/fat accumulates, 137 (11.0%) headache, 128 (10.2%) fatigue/loss of energy, 100 (8.0%) fever/chills or sweats, 95 (7.6%) cough/breathlessness, 89 (7.1%) pain/numbness/tingling in the hands or feet, 87 (7.0%) muscle/joint pain, 72 (5.8%) nausea/vomiting, 59 (4.7%) diarrhoea, 57 (4.6%) abdominal pain, 52 (4.2%) dizziness, 48 (3.8%) sexual dysfunction, 45 (3.6%) loss of appetite/change in food taste, 44 (3.5%) weight loss, 38 (3.0%) sleep disorders, 33 (2.6%) skin problems, 14 (1.1%) memory loss and three (0.2%) hair loss. None of the reported symptoms showed any difference according to gender, even for sexual dysfunction (4.6% in men versus 3.6% in women, *p*=0.41). The frequency of participants reporting at least one self-reported symptom according to follow-up visit was as follows: 244/299 (81.6%) at three months, 19/274 (6.9%) at six months and 18/265 (6.8%) at 12 months. Non-adherence to HAART was reported in 16 (5.1%) of the 344 patients who attended at least one follow-up visit.

#### HRQOL

A significant improvement was noted in patients initiating HAART for both physical and mental aspects of HRQOL after one year. Overall, the mean [standard deviation (SD)] PHS score increased from 45.4 (11.1) at baseline to 60.0 (3.1) at 12 months (*p*<10^−4^) and the mean (SD) MHS score increased from 42.2 (8.7) to 43.9 (3.4) (*p*<10^−2^). Table 2 presents the PHS and MHS scores as well as the eight dimensions of the MOS SF-36 questionnaire at HAART initiation and after the 12-month follow-up visit according to gender. All dimensions of the HRQOL assessed by the MOS SF-36 increased significantly in men and women after 12 months on HAART except for the vitality and general mental health dimensions. Both men and women presented a significant increase in the PHS score over this 12-month period (*p*<10^−4^). The increase in the MHS score was significant in women (*p*<10^−2^) but not in men (*p*= 0.41).

**Table 2:** The Medical Outcome Study 36-Item short-form health scores at HAART initiation and at the 12-month follow-up visit. The IeDEA West Africa collaboration, 2010–2011

	HAART initiation *n*=344 Mean (SD)	12-month visit *n*=265 Mean (SD)	Score change *n*=265 Difference^a^ (CI 95%)	p*
MOS SF-36 dimensions				
Physical functioning				
Women	73.5 (27.2)	98.9 (5.9)	+23.7 (26.6)	<10^−4^
Men	75.4 (25.7)	99.0 (4.8)	+22.7 (26.0)	<10^−4^
Physical-related role limitations				
Women	53.5 (45.7)	99.1 (5.9)	+43.8 (45.5)	<10^−4^
Men	51.9 (45.5)	98.6 (9.5)	+44.9 (47.9)	<10^−4^
Bodily pain				
Women	62.7 (28.9)	97.0 (10.0)	+32.6 (29.2)	<10^−4^
Men	59.1 (26.5)	96.2 (11.3)	+36.3 (25.3)	<10^−4^
General health perception				
Women	60.6 (25.6)	86.0 (12.3)	+24.4 (28.8)	<10^−4^
Men	59.9 (27.4)	88.1 (8.9)	+26.2 (26.9)	<10^−4^
Vitality				
Women	47.6 (17.6)	48.5 (9.4)	+0.5 (19.6)	0.74
Men	47.6 (16.8)	48.0 (8.6)	−0.4 (18.0)	0.87
Social functioning				
Women	71.1 (25.9)	96.4 (8.9)	+23.1 (25.4)	<10^−4^
Men	70.8 (26.4)	96.2 (9.9)	+23.4 (25.3)	<10^−4^
Emotional-related role limitations				
Women	62.7 (43.6)	99.5 (5.3)	+35.7 (43.7)	<10^−4^
Men	62.6 (44.7)	98.6 (12.0)	+33.3 (45.4)	<10^−4^
General mental health				
Women	55.6 (15.5)	54.5 (9.7)	−0.8 (18.4)	0.57
Men	57.9 (12.8)	54.5 (9.0)	−2.7 (15.2)	0.14
MOS SF-36 composite scores				
Physical health summary score				
Women	45.6 (11.2)	60.0 (3.1)	+13.6 (11.4)	<10^−4^
Men	44.9 (10.7)	60.1 (3.2)	+14.6 (11.0)	<10^−4^
Mental health summary score				
Women	42.1 (8.9)	43.9 (3.4)	+1.8 (9.2)	<10^−2^
Men	42.7 (8.0)	43.8 (3.3)	+0.8 (7.9)	0.41

* Matched paired t-test

a mean observed scores differences between 12-month visit and HAART initiation. SD = standard deviation; CI=confidence intervals; MOS SF-36 = Medical Outcome Study 36-Item short form; HAART = highly active antiretroviral therapy.

#### Determinants of HRQOL and their temporal changes

In univariable analysis, a baseline history of discrimination, having disclosed his/her HIV status as well as other baseline measured characteristics, was not significantly associated with MHS or PHS score evolution over 12 months. The type of HAART regimen, experiencing a treatment modification associated with drug toxicity or being identified as non-adherent during follow-up visits, was also not significantly associated with PHS or MHS score evolution in univariable analysis (data not shown). These variables were thus not included in the multivariate model.

Table 3 shows the PHS and MHS mean score change estimated by the multivariable LMM. Although not associated with the PHS score, gender was associated with the MHS score in multivariate analysis. Women presented with a lower baseline MHS score (41.8) compared to men (42.9) (*p=*0.04). Due to a steeper increase in the MHS score in women over time, the 12-month MHS score estimate in women (45.4) was not significantly different compared to the MHS score estimate in men (45.1) (*p*=0.69). The number of perceived symptoms was the main characteristic associated with both PHS and MHS score evolution with an estimated mean decrease for any additional reported symptom of −0.2 (*p*<10^−4^) and −0.1 (*p*<10^−2^) unit/month, respectively.

**Table 3:** Baseline mean physical health summary and mental health summary scores and their mean changes (in units/month) after HAART initiation, estimated by multivariable linear mixed models, the IeDEA West Africa Collaboration, 2010–2011 (344 patients; 1,182 observations)

	Initial PHS score^a^		PHS score change^a^		Initial MHS score^b^		MHS score change^b^	
	
Variables	Mean (95% CI)	*p*	Mean (95% CI)	*p*	Mean (95% CI)	*p*	Mean (95% CI)	*p*
Gender						0.04		0.15
Men	Not estimated^c^		Not estimated^c^		42.9 (41.1;44.8)		+0.2 (−0.1; + 0.5)	
Women	–		–		41.8 (40.1;43.4)		+0.3 (+0.0; + 0.6)	
Age at HAART initiation (years)						<10^−2^		0.10
< 35	Not estimated^c^		Not estimated^c^		42.9 (41.1;44.8)		+0.2 (−0.1; + 0.5)	
≥ 35	–		–		44.5 (42.7;46.3)		+0.0 (−0.2; + 0.3)	
Missing	–		–		41.9 (38.4;45.4)		+0.3 (−0.2; + 0.9)	
Education		<10^−3^		0.02				
No education	41.8 (39.5;44.2)		+2.2 (+1.8; + 2.6)		Not estimated^d^		Not estimated^d^	
Primary	43.8 (41.3;46.4)		+2.0 (+1.6; + 2.4)		–		–	
Secondary and more	44.9 (42.5;47.2)		+1.9 (+1.5; + 2.2)		–		–	
Perceived symptoms	–		–	<10^−4^	–			<10^−2^
For each additional symptom^e^	–		−0.2 (−0.2. −0.1)		–		−0.1 (−0.1; −0.0)	
Baseline WHO clinical stage		<10^−4^		<10^−3^		<10^−2^		0.04
Stage I, II	46.0 (43.2;48.7)		+1.7 (+1.3; + 2.2)		44.8 (42.7;46.9)		−0.0 (−0.3; + 0.3)	
Stage III, IV	41.8 (39.5;44.2)		+2.2 (+1.8; + 2.6)		42.9 (41.1;44.8)		+0.2 (−0.1; + 0.5)	
Missing	43.1 (37.2;49.1)		+2.0 (+1.1; + 3.0)		43.3 (39.1;47.6)		+0.3 (−0.4; + 1.0)	
Baseline CD4 count (cells/mm^3^)		<10^−3^		0.03		<10^−2^		<10^−2^
< 50	41.8 (39.5;44.2)		+2.2 (+1.8; + 2.6)		42.9 (41.1;44.8)		+0.2 (−0.1; + 0.5)	
≥ 50–200	45.5 (43.6;47.3)		+1.7 (+1.5; + 2.0)		43.9 (42.3;45.5)		+0.1 (−0.1; + 0.3)	
≥ 200	47.0 (44.9;49.1)		+1.7 (+1.4; + 2.0)		45.7 (44.0;47.4)		−0.1 (−0.4; + 0.1)	
Missing	48.1 (44.9;51.4)		+1.6 (+1.1; + 2.0)		45.9 (43.4;48.5)		−0.3 (−0.7; + 0.1)	
HIV clinic		<10^−4^		<10^−4^		<10^−4^		<10^−4^
University hospital	41.8 (39.5;44.2)		+2.2 (+1.8; + 2.6)		42.9 (41.1;44.8)		+0.2 (−0.1; + 0.5)	
General public hospital	46.7 (44.2;49.2)		+1.5 (+1.1; + 1.8)		43.0 (41.0;45.0)		+0.2 (−0.1; + 0.5)	
Non-governmental organization	33.6 (31.1;36.0)		+2.9 (+2.5; + 3.3)		35.9 (33.9;37.8)		+0.9 (+0.6; + 1.2)	

a Reference group: no symptoms reported, no education, followed at university hospital, initial CD4 < 50 cells/µl and clinical stage III or IV

b reference group: no symptoms reported, male aged < 35, followed at university hospital, initial CD4 < 50 cells/µl and clinical stage III or IV

c not estimated as the variable was not significantly associated with baseline mean PHS score and/or its mean change in univariate analysis

d not estimated as the variable was not significantly associated with baseline mean MHS score and/or its mean change in univariate analysis

e reported symptoms during follow-up visits were summed for each patient in order to provide a quantitative assessment of the total number of perceived symptoms. CI = confidence interval; HAART = highly active antiretroviral treatment; PHS = physical health summary; MHS = mental health summary; WHO = World Health Organization.

**Figure 1:**
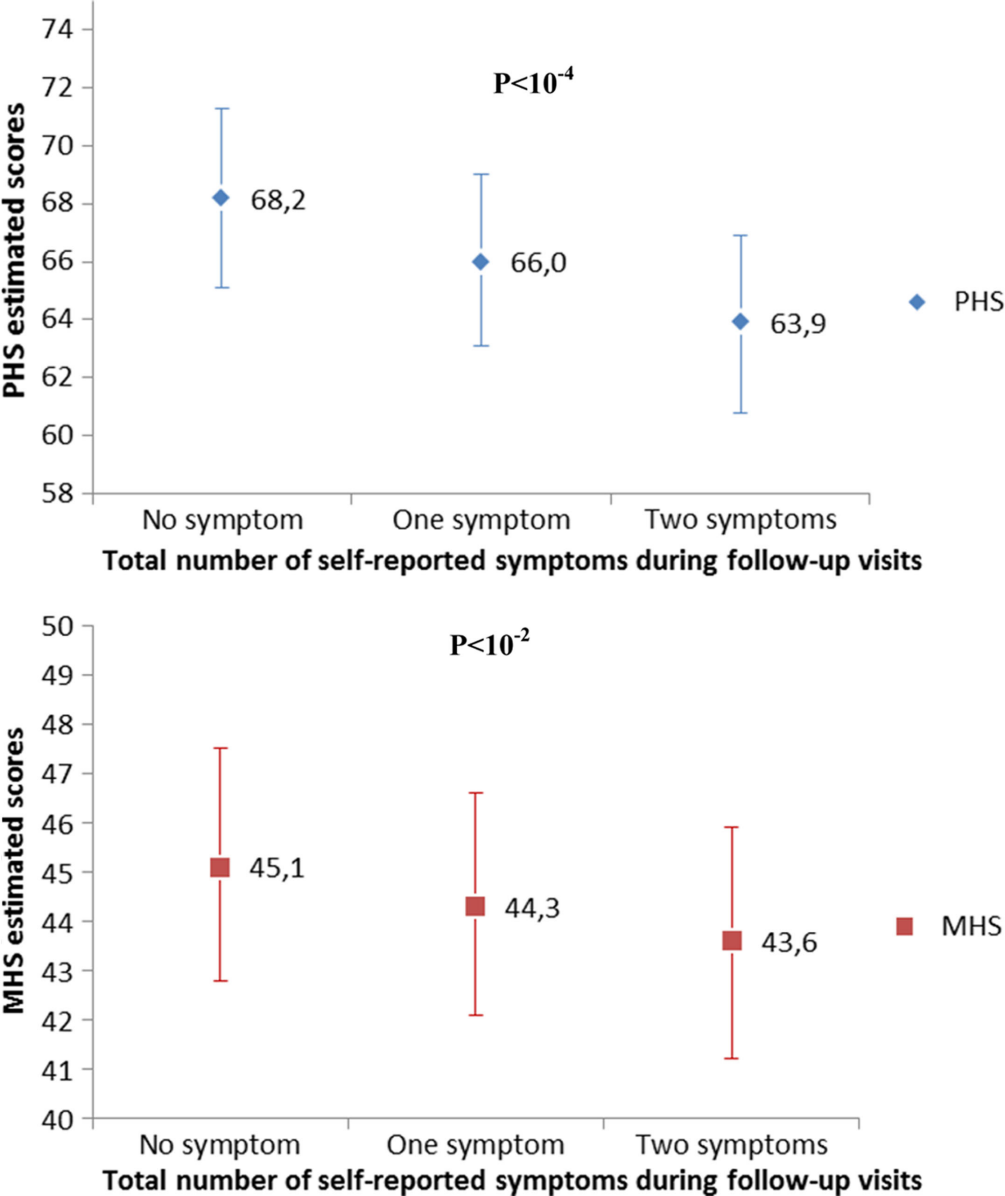
Mean PHS and MHS scores at 12 month after HAART initiation according to the number of perceived symptoms estimated with the multivariable linear mixed models. The IeDEA West Africa collaboration, 2010–2011. PHS = physical health summary; MHS = mental health summary; HAART = highly active antiretroviral treatment.

At 12 months, patients that experienced on average two symptoms per visit presented with significantly lower PHS (63.9) and MHS (43.6) scores compared to patients that presented no symptoms during follow-up with respective PHS and MHS of 68.2 (*p*<10^−4^) and 45.1 (*p*<10^−2^) (Figure 1). Baseline clinical stage and immunological status were also the main determinants of HRQOL changes over a 12-month period. Patients with a WHO stage III/IV presented with a significantly lower PHS (41.8) and MHS (42.9) scores compared to patients with stage I/II with respective PHS (46.0) (*p*<10^−4^) and MHS (44.8) (*p*<10^−3^) scores prior to HAART initiation. However, patients experienced a significant improvement of both PHS and MHS scores irrespective of their baseline WHO clinical stage. At 12 months, PHS and MHS scores were not significantly different between patients that experienced a stage I/II or a stage III/IV clinical outcome prior to HAART initiation with respective *p*-values of 0.14 and 0.48.

The type of HIV clinic was also associated with a significant difference in baseline HRQOL. Patients attending the NGO had lower PHS (33.6) and MHS (35.0) scores compared to patients attending the general public hospital with PHS of 46.7 (*p*<10^−4^) and MHS of 42.1 (*p*<10^−4^). However, the gain in PHS (+2.9 units/month) and MHS (+1.0 units/month) was greater for the patients in care in the NGO-type clinic compared to those followed in the general public hospital who experienced a PHS gain of 1.5 unit/month (*p*<10^−4^) and a MHS gain of +0.3 unit/month (*p*<10^−4^).

## Discussion

A significant increase in both physical and mental aspects of the HRQOL in a population of 344 HIV-positive persons after one year of HAART was measured in Ouagadougou, Burkina Faso. Results of this prospective observational cohort are consistent with previous reports from sub-Saharan Africa and highlight that the use of HAART in real conditions is associated with an improved quality of life in Burkina Faso [9–12]. Moreover, this is the first report from sub-Saharan Africa that includes both perceived symptoms and adherence to HAART in the prospective assessment of HRQOL of HIV-positive persons.

One year of HAART significantly improved most dimensions of HRQOL. All physical dimensions of HRQOL improved, but the impact of treatment was less striking on the mental dimensions, particularly in men for whom the increase in the MHS score was not significant. Women presented with a significantly lower MHS score at baseline compared to men, but a steeper increase was observed after HAART initiation. These results are in accordance with previous findings from high-income settings and emphasize the particular benefit of HAART initiation on women's mental HRQOL [27]. However, such a gender difference has not been reported in previous reports from sub-Saharan Africa so far [10, 11]. Additional studies are needed to confirm these results and explore potential factors associated with gender difference in mental dimensions of HRQOL. Our results also highlight the high level of physical disability and depleted functional autonomy at HAART initiation in sub-Saharan Africa compared to Northern countries. Indeed, results from a French cohort of HIV-positive patients reporting HRQOL evolution one year after HAART initiation showed that physical dimensions of the MOS SF-36 were high at enrolment compared to our results, with mean (SD) physical functioning, bodily pain and physical-related role limitations of 84.5 (21.6) 76.7 (25.2) and 67.8 (38.9), respectively [18]. The relatively low scores in physical dimensions found in our study probably reflect the delay in HAART initiation in Ouagadougou, with patients presenting with more advanced and severe HIV-related diseases.

As reported elsewhere, drug-related toxicity accounts for the majority of treatment modification in HIV-positive patients treated with HAART in sub-Saharan Africa [28, 29]. Previous studies have documented the potential impact of drug-related toxicity on HRQOL. In 2009, Pitt *et al*. reported factors associated with a negative evolution of PHS or MHS scores administrating the MOS SF-36 to 295 HIV-positive patients prior to HAART initiation and 48 weeks thereafter [11]. They found no association between drug-related toxicity and negative PHS or MHS scores. However, they only measured toxicity severe enough to prompt a change in HAART regimen. This could have led to an underestimation of the true negative impact of drug-related toxicity, especially as the treatment withdrawal tends to restore patient's health. In our study, drug-related toxicity confirmed by treatment modification did not have a significant impact on HRQOL evolution in the first year of HAART. However, the number of patient's self-reported symptoms recorded at each follow-up visit was associated with a significant decrease in both physical and mental dimensions of HRQOL over one year. This finding is consistent with previous reports from high-income countries where the number of self-reported symptoms experienced during the first year of treatment among HIV-positive patients initiating HAART was highly predictive of a reduced HRQOL [30, 31]. An appropriate management of reported symptoms during the follow-up of HIV-positive patients initiated on HAART is thus a key component to restore HRQOL. As no causal relation could be formally established between perceived symptoms and drug-related toxicity among HAART-treated patients, there is a clear need to enhance pharmacovigilance activities in a context of HAART scale-up in sub-Saharan Africa. Indeed, previous reports have highlighted the lack of knowledge concerning drug-related adverse events identification and staging in our study environment [28, 32] and elsewhere in Africa [33]. With this respect, appropriate training in the assessment and management of drug-related toxicity is a priority to improve HRQOL.

Despite official recommendations of withdrawal with regards to its known toxicity, stavudine was still largely prescribed as a first-line drug in 2010 in Ouagadougou. Indeed, reported symptoms potentially linked to mitochondrial toxicity (i.e. change in the body image/fat accumulation and pain/numbness/tingling in the hands or feet) were frequently reported by patients during their follow-up visits. Withdrawal of stavudine and sustained access to less toxic recommended first-line and second-line antiretroviral drug regimens are indeed key components for the management of drug-related toxicity, thus maximizing a sustained adherence to HAART and retention in healthcare programs.

After controlling for baseline HRQOL, a baseline-impaired immunological status and the presence of serious HIV-associated disease (stage III or IV WHO classifying disease) were associated with a lower HRQOL prior to HAART initiation but with a subsequent higher increase of HRQOL over 12 months. These results are consistent with prior reports from high-income countries and other settings in sub-Saharan Africa [11, 18, 34]. Some authors have advocated that patients with a less severe baseline immunological and clinical status were more sensitive to the constraints and inconvenience of HAART than to its benefits. This question will require close monitoring as the general tendency is a shift towards a higher CD4 count at HAART initiation.

The type of healthcare facility delivering HAART and HIV services was also a major determinant of baseline HRQOL and its evolution. Indeed, the NGO facility was associated with a significantly lower baseline HRQOL and a subsequent higher increase over 12 months compared to the public sector facilities for both PHS and MHS scores. These differences could be attributed to unmeasured sites characteristics. Many African countries have eliminated user fees for HAART as it was identified as an important barrier to universal access [35]. However, until 2010, Burkina Faso was an exception to this trend of providing HAART for free. Nonetheless, NGO's provided free access to HAART and other healthcare services to selected HIV-positive persons based on the severity of their health condition [36]. This might have selected a specific population attending the NGO site with a particularly low reported HRQOL prior to HAART initiation.

### Limitations

Our study population might not be representative of all HIV-positive patients initiating HAART in Burkina Faso as we essentially included patients living in the urban area of Ouagadougou. However, they might be quite representative of those living in the urban area of Ouagadougou as these three HIV clinics follow the great majority of HIV-positive people of this district. Some patients initially included in the study did not attend the subsequent follow-up visits. These patients might have presented a quite different of HRQOL during their follow-up, introducing potential biases in our PHS and MHS estimates. However, the comparisons of baseline characteristics of patients that attended the three following visits versus the ones that missed at least one visit did not show any significant differences in terms of age, baseline CD4 count and clinical stage (data not shown). Our modelling approach that allowed the inclusion of all patients as long as they had been seen at a baseline provided more accurate estimates of HRQOL. Like previous reports assessing HRQOL in HIV-positive persons, the positive effect of HAART was only documented for the first 12-month period. Further studies are now needed to explore the sustainability of HRQOL after one year of HAART exposure. Finally, as there is currently no MOS SF-36 form that has been validated in the general population of Burkina Faso, we do not have any “normal level” score of the PHS and MHS to refer to. There is now a need to develop culturally adapted and validate tools to assess HRQOL in countries of sub-Saharan Africa.

## Conclusions

The initiation of HAART was associated with a significant improvement in both physical and mental dimensions of HRQOL in HIV-positive patients over a one-year period in Burkina Faso. Despite a lower baseline HRQOL, patients with a low immunological status and more advanced clinical stage presented a steeper HRQOL increase showing that HIV-positive patients presenting with a severe clinical condition can achieve similar HRQOL recovery compared to patients with less severe condition at HAART initiation. Although no association was found between HRQOL and drug-related toxicity inducing treatment modification, the number of symptoms reported by patients taking HAART was associated with a lower HRQOL increase over 12 months. Particular attention must be paid to the identification and management of self-reported symptoms to enhance HRQOL.
